# Developmental Differences in Neuromagnetic Cortical Activation and Phase Synchrony Elicited by Scenes with Faces during Movie Watching

**DOI:** 10.1523/ENEURO.0494-21.2022

**Published:** 2022-05-05

**Authors:** Nataliia Kozhemiako, Adonay S. Nunes, Alexander Moiseev, Amparo V. Márquez-García, Teresa P.L. Cheung, Urs Ribary, Sam M. Doesburg

**Affiliations:** 1 Biomedical Physiology and Kinesiology, Simon Fraser University, Burnaby, British Columbia V5A 1S6, Canada; 2 Department of Psychiatry, Brigham and Women's Hospital, Harvard Medical School, Boston, Massachusetts 02115; 3 Department of Neurology, Massachusetts General Hospital, Harvard Medical School, Boston, Massachusetts 02114; 4 Behavioral & Cognitive Neuroscience Institute, Simon Fraser University, Burnaby, British Columbia V5A 1S6, Canada; 5 School of Engineering Science, Simon Fraser University, Burnaby, British Columbia V5A 1S6, Canada; 6 Department Pediatrics and Psychiatry, University of British Columbia, Vancouver, British Columbia V6T 1Z4, Canada; 7 BC Children’s Hospital Research Institute, Vancouver, British Columbia V5Z 4H4, Canada; 8 Department of Psychology, Simon Fraser University, Burnaby, British Columbia V5A 1S6, Canada

**Keywords:** naturalistic viewing, magnetoencephalography, face processing, development, time-locked activation

## Abstract

The neural underpinnings of humans’ ability to process faces and how it changes over typical development have been extensively studied using paradigms where face stimuli are oversimplified, isolated, and decontextualized. The prevalence of this approach, however, has resulted in limited knowledge of face processing in ecologically valid situations, in which faces are accompanied by contextual information at multiple time scales. In the present study, we use a naturalistic movie paradigm to investigate how neuromagnetic activation and phase synchronization elicited by faces from movie scenes in humans differ between children and adults. We used MEG data from 22 adults (6 females, 3 left handed; mean age, 27.7 ± 5.28 years) and 20 children (7 females, 1 left handed; mean age, 9.5 ± 1.52 years) collected during movie viewing. We investigated neuromagnetic time-locked activation and phase synchronization elicited by movie scenes containing faces in contrast to other movie scenes. Statistical differences between groups were tested using a multivariate data-driven approach. Our results revealed lower face-elicited activation and theta/alpha phase synchrony between 120 and 330 ms in children compared with adults. Reduced connectivity in children was observed between the primary visual areas as well as their connections with higher-order frontal and parietal cortical areas. This is the first study to map neuromagnetic developmental changes in face processing in a time-locked manner using a naturalistic movie paradigm. It supports and extends the existing evidence of core face-processing network maturation accompanied by the development of an extended system of higher-order cortical areas engaged in face processing.

## Significance Statement

While the bases of development of face processing are well investigated using standard paradigms, much less is known about the developmental differences when a more complex and ecologically valid paradigm is applied. In the present study, we investigated developmental changes in whole-brain time-locked activation and connectivity elicited by movie scenes with faces. Our findings indicate reduced activation in core face-processing areas in children compared with adults during the 120–330 ms after stimulus onset. Whole-brain connectivity was also decreased in children in theta and alpha frequency bands between primary visual areas but also extended to higher-order cortical regions in frontal and parietal lobes. Our results support the feasibility of exploiting naturalistic stimuli to investigate specific questions regarding sensory processing as an alternative to standard paradigms.

## Introduction

The ability to process faces efficiently is crucial for everyday social interactions and is supported by a specialized system of brain areas in the occipital and temporal lobes. There is evidence that this system might be genetically predetermined, but it is also shaped by the experience of viewing faces in daily life (Sasson, 2006). Multiple studies investigated the development of behavioral abilities related to face processing as well as the neural mechanisms supporting it. Extensive evidence suggests that humans are highly skilled in recognizing and identifying faces and reading various facial characteristics such as emotional expressions and social intentions (for review, see Young and Burton, 2018). Neuroimaging studies have identified the face-processing network with its three core regions including middle fusiform gyrus (FG), lateral inferior occipital gyrus [occipital face area (OFA)], and the posterior part of superior temporal sulcus (pSTS; Kanwisher and Yovel, 2006; Haxby and Gobbini, 2011). Electrophysiological research has uncovered the temporal specifics of face processing using event-related potentials (ERPs) or event-related fields (ERFs) in MEG studies leading to the identification of the face-sensitive components—N170/M170, P250/M250, P400/M400 (for review, see Eimer, 2011; Marzi and Viggiano, 2007; Puce et al., 2013).

Early electrophysiological studies demonstrated profound developmental differences in amplitudes and latencies of P100, P170, and M250, and the adult-like face-related brain responses can be observed only in late childhood (Taylor et al., 2004; He and Johnson, 2018). Large-scale investigation of changes in cortical activation and connectivity dynamics during brain development reported activation in face-selective cortical areas together with a nonspecific activation in other brain regions (Joseph et al., 2011) and that face selectivity and modular activation of the core face-processing regions increased with age (Haist et al., 2013). Another study reported a decrease in information transfer within the face network in adults compared with children, potentially reflecting an increase in specialization between parts of the network. (Joseph et al., 2019). Multiple studies have also reported lower connectivity within the core face network in children compared with adults, and that connectivity strength within this network increased with age (Kadosh et al., 2011; Joseph et al., 2012; Song et al., 2015; He et al., 2015a). Together, these findings suggest large-scale configurational changes in face processing from childhood to adulthood.

While the bases of the development of face processing are well investigated using standard paradigms, much less is known about the developmental differences when a more complex and ecologically valid paradigm is applied. Traditional paradigms usually consist of a set of standardized pictures of faces that are repeatedly presented to a viewer to elicit brain activation in brain areas specialized for face processing. The advantage of such paradigms is that a researcher can control basic physical properties of the stimuli and manipulate different aspects such as emotional expressions, similarity, and positioning of the faces. Such increased experimental control comes at the expense of ecological validity, however, as the studied stimuli are oversimplified, isolated, and decontextualized (Wieser and Brosch, 2012). An alternative approach—naturalistic viewing—is becoming more common. Instead of repeated simple stimuli, participants are presented with a movie that resembles closely the complexity of sensory information we are exposed to in real life. Apart from increased ecological validity, naturalistic viewing paradigms are easier to conduct when collecting data in challenging populations such as children and participants with neuropsychiatric or neurologic conditions (Vanderwal et al., 2015; Greene et al., 2018). Recently, scenes from a movie were used to do a time-locked analysis to investigate the neuromagnetic dynamics of visual processing and to map face-selective brain responses (Nunes et al., 2020).

In this study, we used MEG and a data-driven multivariate statistical approach to investigate time-locked neuromagnetic activation and phase synchrony between cortical areas in response to scenes with faces in children and adults during movie watching. Rather than focusing on traditional univariate parameters of event-related responses constricted to a specific area, we examined whole-brain activity and synchronization to capture complex and widespread patterns of cortical activation that could be produced by naturalistic stimuli. To target activation specific to faces, we use adopted an approach that contrasts the activation pattern elicited during the scenes with faces to the activation elicited during other scenes with no faces on the screen. Based on prior literature using standard paradigms, we expected to observe increased activation in core face-processing regions as well as increased connectivity within this network in adults compared with children. We also hypothesized that the areas from the extended face-processing network would also display developmental differences in face-elicited activation and connectivity as their involvement is needed for efficient processing and integration of rich incoming information conveyed by faces and the surrounding background of movie scenes.

## Materials and Methods

### Participants

MEG data were recorded from 22 adults (6 females, 3 left handed; mean age, 27.7 years; SD, 5.28 years) and 20 children (7 females, 1 left-handed, mean age, 9.5 years; SD, 1.52 years). A detailed description of the cohort is provided in Table 1 (the details regarding statistical comparison results provided in the table are described in the statistical analysis section below). None of the participants reported any psychiatric or neurologic conditions. All participants had normal or corrected-to-normal vision and reported no hearing problems. Informed consent was obtained from all participants. Additionally, informed consent was obtained by parents or legal guardians of child participants. This study was approved by the Research Ethics Board of Simon Fraser University.

**Table 1:** The cohort characteristics

Variables	Adults	Children	*p*-values
Participants, *n*	22	20	
Females/males, *n*	6/16	7/13	*p* = 0.84
Age (years)	27.7 (5.28)	9.5 (1.52)	*p* < 0.001
Age range (years)	21–42.5	7.2–12.9	
Handedness (right/left), *n*	19/3	19/1	*p* = 0.67
Head circumference	56.6 (1.9)	54.6 (1.44)	*p* < 0.001
Epochs included in the analysis, *n*	102 (0.2)	100 (6.7)	*p* = 0.16
Head movement, mm	0.7 (0.35)	1.5 (1.03)	*p* < 0.001

Values are mean (SD), unless otherwise indicated.

### MEG data collection

MEG data were collected using a 275-channel MEG system (CTF MEG Neuro Innovations) in an AK3B magnetically shielded room (Vacuumschmelze) at a sampling frequency of 1200 Hz. During data collection, participants viewed a short movie clip projected onto an MEG-compatible screen 40 cm above their eyes while lying in the supine position. The movie audio was presented via MEG-compatible earphones, which were inserted before the data collection, and a short test was conducted to ensure that each participant could hear sound clearly in both ears. Each participant viewed the movie clip twice with a short break between the presentations. All participants were instructed to pay attention to both movie presentations and to try to keep as still as possible. Head position in the dewar was recorded using three fiducial coils placed at the nasion and left and right preauricular locations. To enable coregistration between the MEG and structural MR image coordinate systems, each participant’s head shape and fiducial coil locations were digitized with a FASTRAK digitizer (Polhemus) before the MEG data collection.

### MRI data collection

Most of the participants (22 adults and 18 children) attended the second session of data collection when a T1-weighted anatomic image was collected using a Philips 3T Ingenia CX scanner with the following settings: TE = 3.7 ms; flip angle = 8°; FOV = 256 × 242; matrix size = 256 × 242; slice thickness = 1 mm; number of slices = 213; sagittal orientation. Two child participants, however, did not return for the second session of data collection; thus, the T1-weighted image was not available for them. For these two participants, we selected the best matched MRI from the pool of the other 18 participants based on the mean distance between each Polhemus point and the closest point on the skull surface derived from the segmented T1 image using the Fieldtrip toolbox (Oostenveld et al., 2011). The same procedure was previously successfully applied in the children cohort of similar age (Kozhemiako et al., 2019, 2020).

### Stimulus paradigm

**Figure 1.**
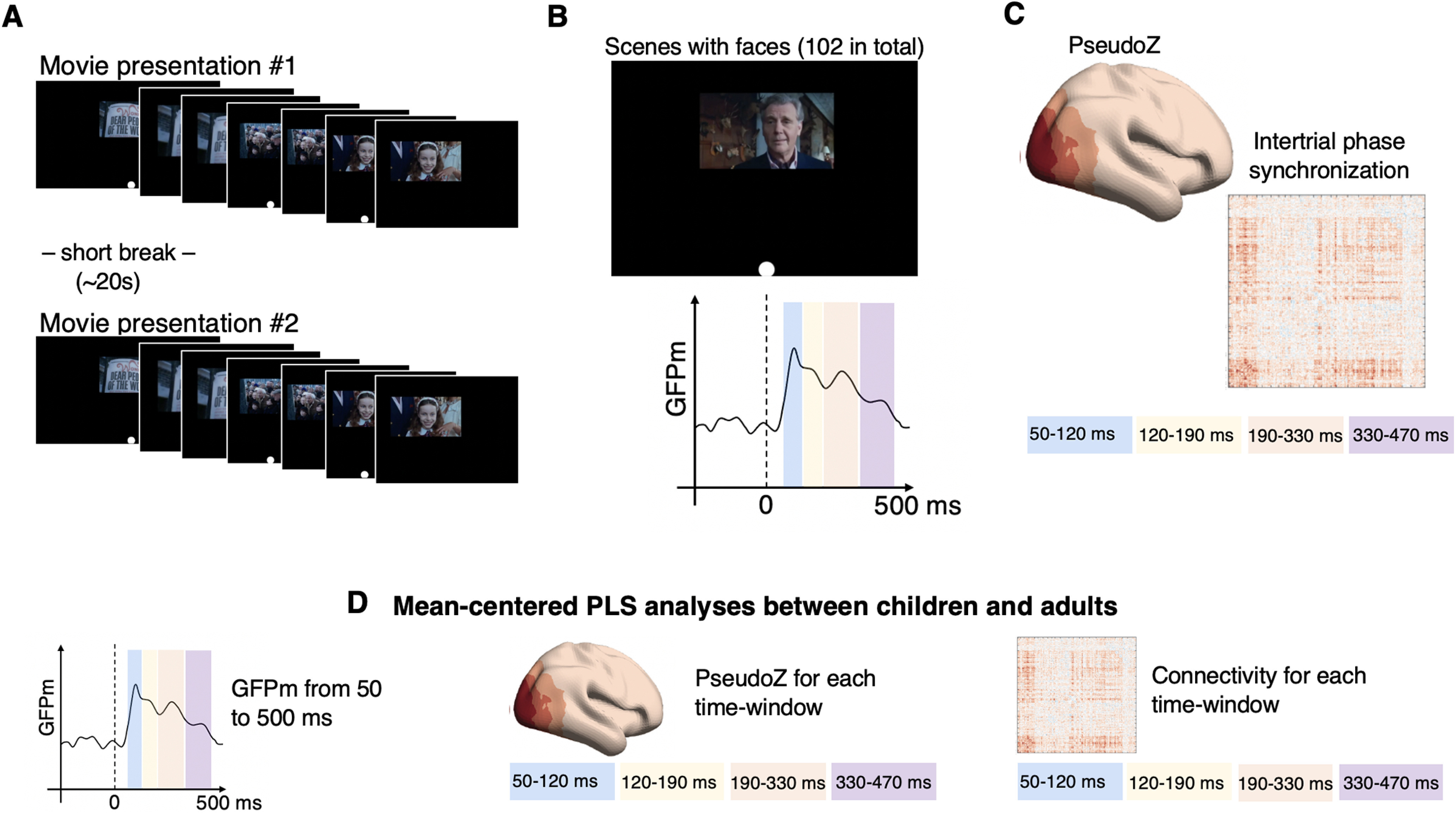
Schematic illustration of study design and analysis workflow. ***A***, Two movie presentations (4 min, 32 s each) composed of scenes from “Charlie and the Chocolate Factory” (2005) were presented to children and adult participants with a short break in between. During movie watching, MEG data were collected for each participant. We also recorded a photodiode signal registering the change in luminance at each new scene because of the white dot on the black background placed on the first five frames of each new scene. ***B***, Scenes containing faces (51 faces per movie clip; total, 102 faces) were extracted, and the photodiode signal was used to define the 1000 ms epochs (prestimulus interval, 500 ms; poststimulus interval, 500 ms) with time at zero indicating the onset of face scenes. Subsequently, epochs were used to compute GFPm separately for children and adults. GFPm peaks were later used to define the time windows of interest. ***C***, For each of the time windows of interest, we computed PseudoZ to assess cortical activation elicited by face scenes and intertrial phase synchronization to evaluate cortical connectivity time locked to face scene onset. ***D***, Resulting measures were tested for significant differences between children and adult participants.

The movie clip presented during MEG recording was 4 min and 32 s long and consisted of scenes from the movie “Charlie and the Chocolate Factory” (2005) directed by Tim Burton. Here we used “movie frame” as a term to describe a single image in a movie 24 frames/s) and “movie scene” to denote a movie segment (minimum of 15 movie frames long but usually lasting several seconds) composed of some number of similar frames. The movie clip was manually divided into scenes for the purpose of further scene classification. In addition, to ensure the precision of time-locked analysis, the first five frames of each scene were marked by a white circle that would appear on the black background (Fig. 1*B*). A photodiode was fixed at the location on the screen where the white circle was projected during movie presentation to detect changes in luminance. The photodiode signal was used to identify the precise time when each scene appeared on the screen. Each movie scene was manually classified into different categories which included the scenes containing primarily faces (51 occurrences). For the time-locked analysis, we combined the scenes from both movie presentations and used the time when each scene with a face appeared on the screen to define the epochs (102 1-s-long epochs in total). For PseudoZ calculation, other scenes picturing hand manipulations, distant human bodies, scenery, and nonliving objects (78 in total) were used to define epochs based on which the noise covariance was computed (described in detail in the PseudoZ subsection below). In addition, we conducted a control analysis to rule out the possibility that differences between groups were produced by some artifact shared between different conditions (e.g., signal leakage because of volume conduction). We used scenes with hands manipulations (58 in total) to define epochs for the control analysis.

### Data preprocessing

To control for the effects of excessive head movement, time points with movement exceeding a 10 mm threshold (in any direction for any of the three fiducial coils) were detected, and the epochs that overlapped in time with excessive movement were excluded from the analysis. For the included epochs, the median of head movement was computed for each participant to investigate potential group differences. Next, the data were visually inspected to identify and exclude bad channels. A bandpass filter from 1 to 150 Hz was applied to the data, and line noise was removed by applying a notch filter at the power line frequency of 60 Hz and harmonics. Independent component analysis was applied, and components composed of oculographic (e.g., blinks, saccades) and cardiac artifacts were removed after visual inspection. One second epochs were later extracted based on the stimulus onset timing spanning a 0.5 s prestimulus interval and a 0.5 s poststimulus interval.

### Sensor level time-locked responses

We investigated sensor level time-locked responses to scenes with faces to define the time windows of interest for whole-brain activation and phase synchronization analysis. The Fieldtrip toolbox was used to low-pass filter at 40 Hz and downsample the epochs to 300 Hz to reduce computational load for the statistical analysis (described below; Oostenveld et al., 2011). Then the epochs time locked to the stimulus onset were averaged in children and adults. A magnetic counterpart of global field power, global field power magnetic (GFPm) was computed as a root mean square across all MEG sensors and baseline corrected using the time interval from −200 ms to stimulus onset.

### Source modeling

The T1-weighted MR image of each participant was realigned based on the coordinates of fiducial coils and segmented to extract cortical, skull, and scalp surface using the Fieldtrip toolbox. A single-shell head model was generated based on each participant’s inner skull surface. The geometry of cortical surface for each participant was reconstructed using Freesurfer, resulting in a cortical mesh of 4002 vertices per hemisphere on the standard space (Fischl et al., 1999). The forward solution was generated for each vertex from the cortical mesh using the Fieldtrip toolbox.

### PseudoZ calculation

**Figure 2.**
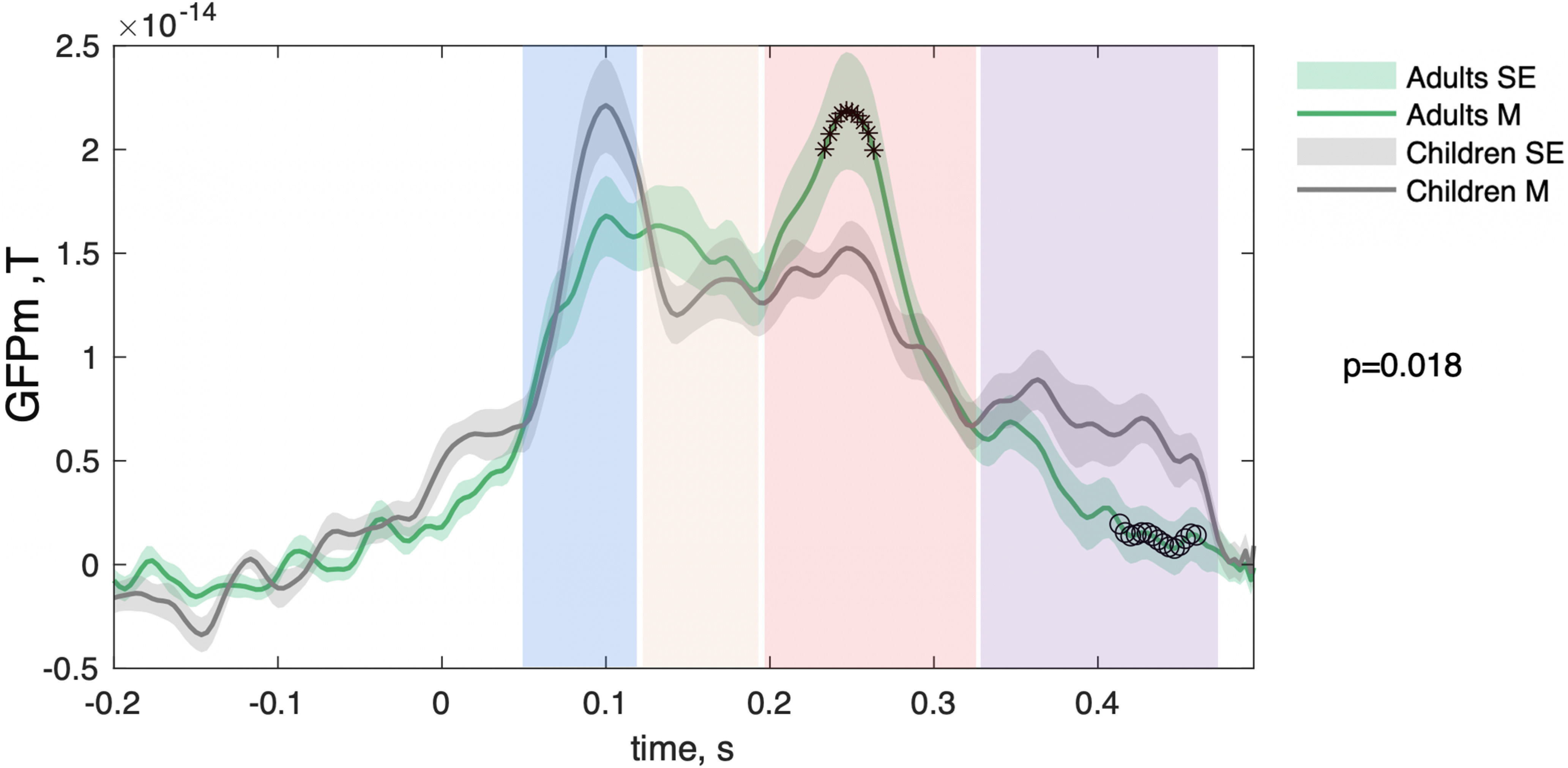
Difference in the time-locked responses to face scenes during movie watching between child and adult groups. The PLS analysis of GFPm across time points from 50 to 500 ms indicated significant group differences (*p* = 0.018). GFPm in adults (in green) and children (in gray) were averaged across all MEG channels and face epochs. The shadow represents the SE, and star markers illustrate time points where adults had higher GFPm values compared with children based on PLS *z* score threshold (*z* score, >2), whereas circle markers represent the opposite (*z* score, < −2). The rectangular shaded areas illustrate the time windows chosen for further analysis: 50−120, 120−190, 190−330, and 330−470 ms.

As a proxy to brain activation, we computed PseudoZ for each vertex on the cortical mesh using a scalar beamformer for sources oriented orthogonal to the cortical surface (but see Nunes et al., 2020). Essentially, PseudoZ reflects the ratio of event-related source power at a given time to the source power at time of no interest. In our case, the time of interest was defined as 0–500 ms of all epochs when faces were displayed on the foreground (102 in total). Noise covariance was computed based on other epochs, including distant bodies, scenery, and nonliving objects (78 in total). Such an approach essentially contrasts the activation pattern elicited during the scenes with faces to the activation elicited during other scenes with no faces on the screen. Alternatively stated, PseudoZ values will be high only if the activation was specific to scenes with faces and was not present during other scenes. To estimate dynamic changes in evoked activation, PseudoZ was computed for four time windows of interest delineated based on visual inspection of GFPm peaks observed in both groups (Fig. 2) and previous literature reporting the most common components elicited by face stimuli (Puce et al., 2013). This resulted in the following time windows of interest: 50–120 ms approximately corresponding to the M100 component; 120–190 ms approximately corresponding to the M170 component; 190–330 ms approximately corresponding to the M250 component; and 330–470 ms approximately corresponding to the M400 component, consistent with previously reported ERFs elicited by face stimuli.

### Source reconstruction and intertrial phase synchronization

Using a scalar beamformer, we computed spatial filters to reconstruct the time series of each vertex (see Nunes et al., 2020). For the purpose of dimensionality reduction, we used the regions of interest (ROIs) defined by the MultiModal Parcellation Atlas (MPA) to compute intertrial phase synchronization (Glasser et al., 2016). In total, MPA outlines 362 cortical areas. In our analysis, we included 360 cortical areas excluding a medial wall ROI in both hemispheres. Note that although the number of ROIs exceeds the number of channels, in our analysis we do not set an assumption that the signals in ROIs are linearly independent. Glasser et al. (2016) also provided the list of 22 groups encompassing ROIs with similar location, anatomic, and functional properties. We use these groups to label the ROIs (see Figs. 4, 5). Those include the following: primary visual cortex (PVC); early visual cortex (EVC); dorsal stream visual cortex (DSVC); ventral stream visual cortex (VSVC); MT+ complex and neighboring visual areas (MT); somatosensory and motor cortex (SMS MOTOR); paracentral lobular and mid-cingulate cortex (PCL MCC); premotor cortex (PMC); posterior opercular cortex (POC); early auditory cortex (EAC); auditory association cortex (AAC); insular and frontal opercular cortex (Ins FOC); medial temporal cortex (MTC); lateral temporal cortex (LTC); temporo-parieto-occipital junction (TPOJ); superior parietal cortex (SPC); inferior parietal cortex (IPC); posterior cingulate cortex (PCC); anterior cingulate and medial prefrontal cortex (ACC MPC); orbital and polar frontal cortex or PFC; inferior frontal cortex (IFC); and dorsolateral prefrontal cortex (DLPFC).

Before sample phase-locking value (sPLV) calculation, each ROI time course was estimated by averaging the time courses of all vertices within the ROI boundaries accounting for arbitrary polarity as described in the study by Nunes et al. (2020). Then spectral decomposition using a continuous wavelet transform centered at 15 frequency bins spaced on a logarithm scale from 4 to 40 Hz (we decided to avoid high gamma frequency because of its high sensitivity to muscle artifacts) was performed, and sPLV between the signals of ROI pairs was computed for each sample from –200 to 500 ms as follows:

$${\text{sPLV}}_{i,j,t,f}=\frac{1}{N}\overset{N}{\underset{k=1}{\sum }}e^{\Delta \phi_{x,y\left(t,f\right)}},$$

where *N* is the number of epochs, and Δϕ is the phase difference between *x* and *y* at time *t* and frequency *f*. As in the PseudoZ analysis, sPLV was then averaged within four time windows (50–120, 120–190, 190–330, and 330–470 ms) and baseline corrected (baseline interval from −200 to stimulus onset) to remove non-task-related spurious synchronization.

### Statistical analysis

Mean-centered partial least squares (PLS) analysis (McIntosh and Lobaugh, 2004; Krishnan et al., 2011) was selected to perform statistical testing of group differences between children and adult participants because of its numerous advantages. First, it is a multivariate approach that considers multiple features at once when testing group differences and produces a single *p*-value providing a method for addressing multiple comparisons in a manner well suited for neuroimaging studies. Second, it is very tolerant of high collinearity in data that is characteristic of neuroimaging. Third, PLS uses permutation and bootstrapping techniques to test the significance and robustness of the results and, thus, can be used even if the data do not meet the assumption of normality. Fourth, there is no need to specify a predefined group contrast when using the mean-centered PLS as it generates a data-driven group contrast fully based on existing data. In our analysis, we used PLS toolbox (version 6.15.1) implemented in MATLAB (https://www.rotman-baycrest.on.ca/index.php?section=84).

**Figure 3.**
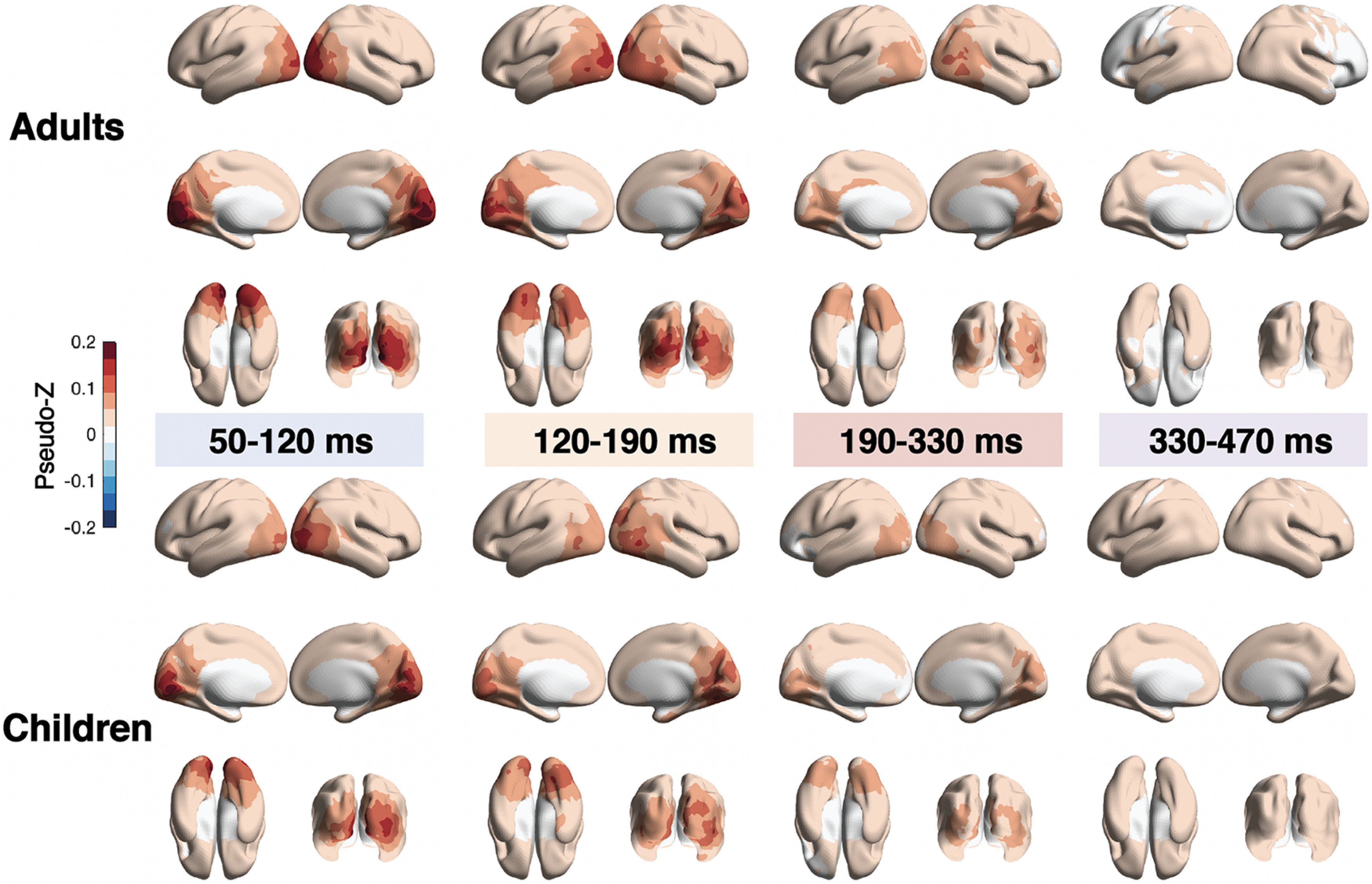
Spatial distribution of brain activation induced by faces during movie watching in adults and children. PseudoZ was averaged across four time windows (50−120, 120−190, 190−330, and 330−470 ms), which were defined based on the time-locked responses illustrated in Figure 1*A* (please note that this is a qualitative illustration, and PseudoZ values are not statistically thresholded).

**Figure 4.**
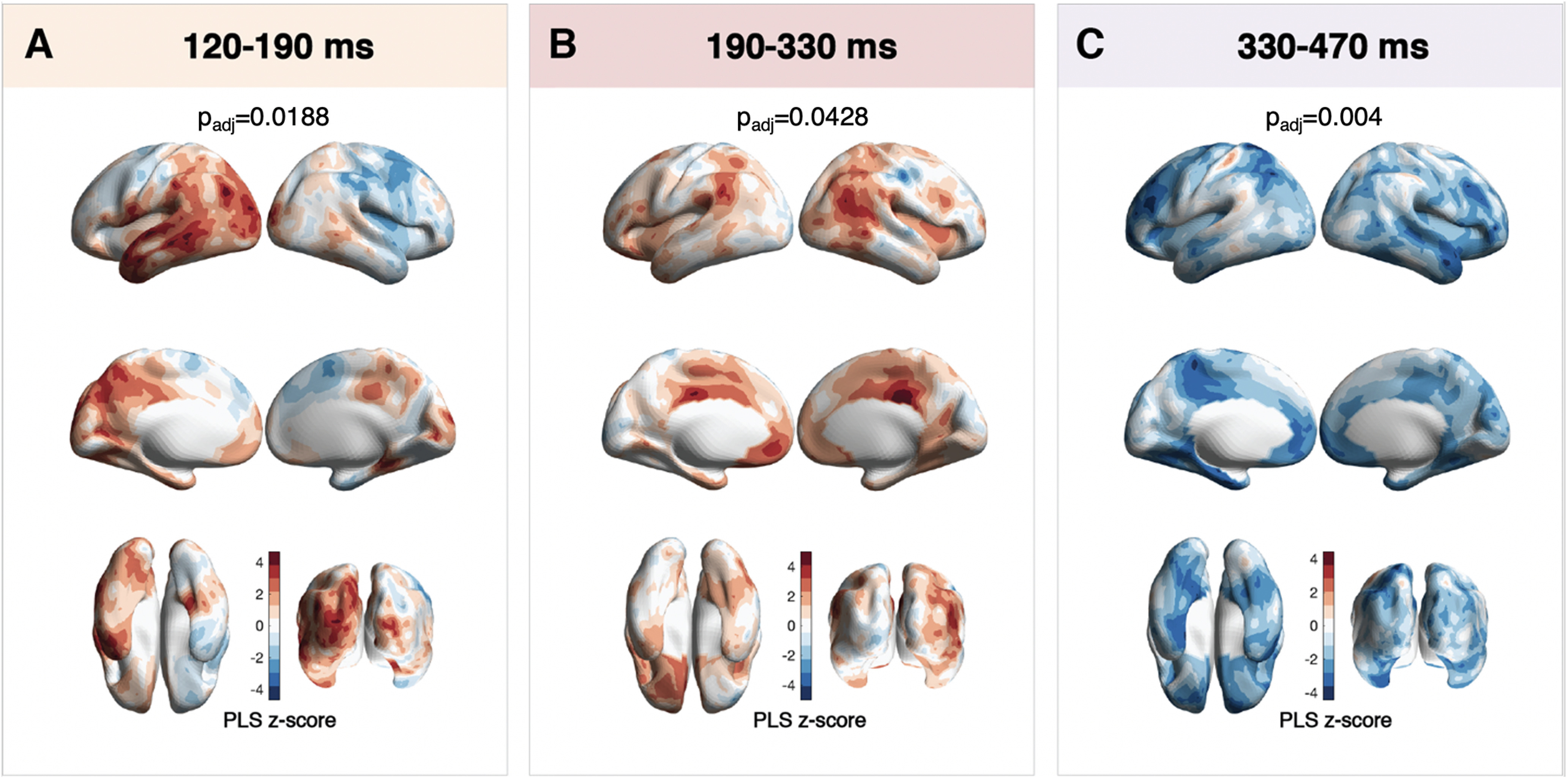
Differences in PseudoZ activation elicited by faces during movie watching in adults versus children. ***A***, PLS *z* score distribution during the 120−190 ms time window. ***B***, PLS *z* score distribution during 190−330 ms time window. ***C***, PLS *z* score distribution during the 330−470 ms time window. Areas in red correspond to the positive *z* score, meaning higher activation in adults compared with children. Areas in blue correspond to negative *z* score and represent higher activation in children compared with adults. Padj – PLS *p*-value adjusted for multiple comparisons.

**Figure 5.**
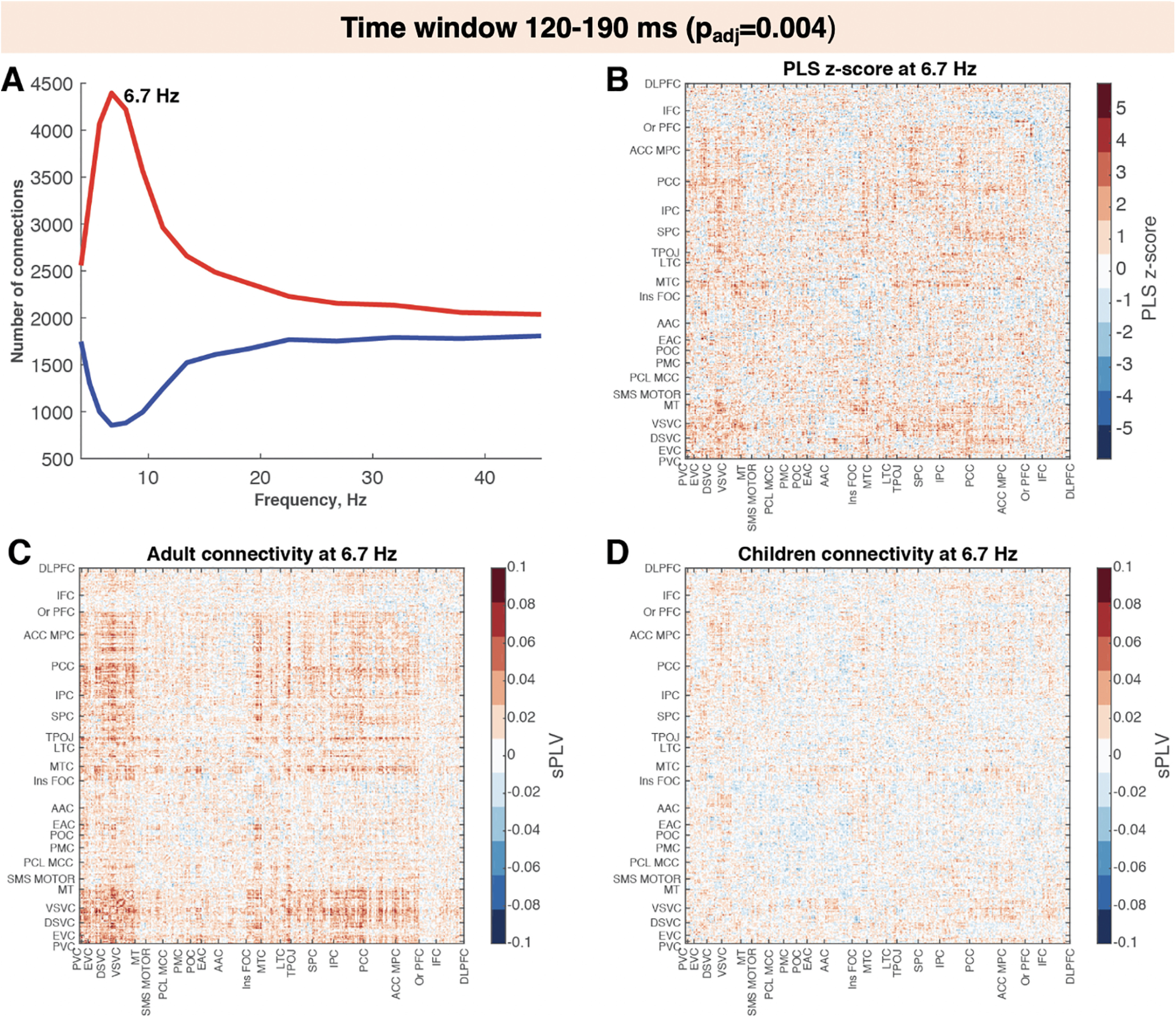
Adults display higher intertrial synchrony compared with children while viewing faces during the 120−190 ms time window. ***A***, Number of connections with the highest (>2, in red) or lowest (less than −2, in blue) *z* score across frequency bins. ***B***, The *z* score distribution across connections for frequency bin with the highest number of connections with *z* score above the selected threshold (6.7 Hz). ***C***, ***D***, Connectivity matrix for adults (***C***) and for children (***D***) at the same frequency bin.

PLS analysis uses singular value decomposition (SVD) to extract latent variables (LVs), which express the most variance in the data. For mean-centered PLS, data are organized in a 2D matrix (number of participants × features) for each group (children and adult participants). Subsequently, the data matrices are mean centered and averaged across the participants of each group, resulting in two vectors. They are then transposed and combined into a 2D matrix (features × number of groups) that is subjected to SVD. That is why the number of LVs is always equal to the number of groups for mean-centered PLS. Each LV is composed of the following three elements. (1) The first is a singular vector that represents a data-driven group contrast. (2) The second is a singular value that indicates the total variance of the data expressed by this LV. The latter is used to test the LV significance using a global permutation test, as follows. First, a null distribution is created by randomly permuting participants between the group data matrices, mean-centering and averaging the permuted group data matrices. Then SVD is performed to extract LVs based on permuted data, and their singular values are saved. After repeating the above steps, *n* number of times (in our case, we used 3000 permutations for each PLS analysis), the *p*-value for the original un-permuted data is computed based on how many randomly generated singular values were larger than the original singular value divided by the number of permutations. In other words, we compute the probability of observing an LV based on a randomly permuted sample that captures the same or more variance as the LV of the original sample. (3) The third is that a singular vector is produced that expresses SVD loadings for each data feature (e.g., time points for GFPm, vertices for PseudoZ, and pairs of ROIs for PLV). Series of bootstraps were used later to generate bootstrap ratios for each data feature that demonstrate the contribution of each data feature to the group contrast and could be interpreted as a *z* score. Specifically, we used 3000 bootstrapping rounds for each PLS analysis. In the Results section, we report spatial distribution of *z* scores on the brain surface (Fig. 3) or as a connectivity matrix (Figs. 4*B*, 5*B*). The *z* score threshold of −2 and +2 approximately corresponds to the 95% confidence interval (McIntosh and Lobaugh, 2004).

In total, nine PLS tests were run for the face-processing analysis. We used a single PLS analysis to compare the GFPm at each time point of the poststimulus time interval between 50 and 500 ms between children and adults. We used a separate PLS analysis for each time window (i.e., four altogether) to compare PseudoZ between groups at all vertices. Similarly, four PLS analyses were performed testing connectivity differences between groups during four time windows. The same number of PLS tests were run for the control analysis. Although we had two LVs for each PLS test as a result of two group comparison, in our analyses we always focused on the first LV because it explained the largest proportion of variance. To account for multiple PLS comparisons, the *p*-values for PseudoZ activations and connectivity comparisons were adjusted for multiple comparisons using the Bonferroni method.

To test whether male/female or left-handed/right-handed participant proportions were different between children and adult samples, we used a χ^2^ test. To test whether there were differences in age, head size, head motion, and the number of included epochs, we used a two-tailed *t* test. The results are presented in Table 1. We also used a two-tailed t-test to perform a univariate analysis of GFPm across all channels averaged within 50-120 ms and 120-190 ms to target M100 and M170, respectively.

## Results

We first investigated event-related neuromagnetic activation at the sensor level. To this end, we computed the GFPm across all MEG sensors and averaged it across face epochs in every individual. The results were further averaged across individuals separately for each group, and the corresponding waveforms are illustrated in Figure 2. To test whether GFPm were significantly different between children and adults, we ran a PLS analysis. The comparison revealed significant differences between children and adults (*p* = 0.018). To identify the time points when differences between groups were the most robust, we used a *z* score threshold of 2 and −2. We plotted time points with *z* scores >2 with star markers indicating higher GFPm values in adults and time points with *z* scores less than −2 with circle markers, which can be interpreted as higher GFPm in children compared with adults. Based on PLS *z* scores, adults had higher GFPm values during the 230–260 ms that correspond to the M250 component. In contrast, children exhibited increased GFPm values between 410 and 460 ms compared with adults. In addition, we conducted a univariate analysis targeting the classic ERFs. To do so, we averaged GFPm from 50 ms to 120 ms (M100) and from 120 ms to 190 ms (M170). No significant differences (*p* < 0.05) were observed for M100 or M170 whether raw or normalized by the corresponding segments of GFPm computed based on the hand scenes.

### PseudoZ activation elicited by faces in children and adults

Based on GFPm waveform peaks, we defined four time windows of interest (50–120, 120–190, 190–330, and 330–470 ms) for which PseudoZ was computed to assess the spatial distribution of time-locked activation in response to face stimuli. PseudoZ activation for each time window averaged for children and adults is plotted on the brain surface in Figure 3. During the first time window (50–120 ms) in both groups, the PseudoZ activation was located in the occipital lobe corresponding to primary and early visual cortex. During the second time window (120–190 ms) the activation protracted in the lateral and inferior direction following the ventral visual stream. During the third and fourth time windows, the PseudoZ values started to diminish in both groups. In adults, however, during 190–330 ms time window PseudoZ activation was still visually apparent lateralized to right hemisphere in lateral occipital cortex and the posterior part of the superior and middle temporal gyri. A similar pattern was absent in child participants.

To assess whether there were significant differences in PseudoZ activation between children and adults, we ran four separate PLS analyses (one for each time window). The comparison of all but the first time window (*p*_adj_ = 0.76) revealed significant differences between children and adults in the time-locked cortical activation in response to face appearances on the screen. During the time period of 120–190 ms, PseudoZ activation in adults was higher (*p*_adj_ = 0.0188) in the occipital, parietal, and temporal lobes of the left hemisphere (Fig. 4*A*). More precisely, the areas with the highest *z* scores included lateral occipital, inferior parietal areas as well as middle and superior temporal gyri. Children, however, expressed higher activity in right marginal gyrus and right frontal cortical areas corresponding to the posterior part of middle frontal gyrus and the frontal eye field.

PLS comparison of PseudoZ activation at 190–330 ms also revealed significant differences (*p*_adj_ = 0.0428) between child and adult participants (Fig. 4*B*). Higher time-locked activation was observed in adults in right pSTS, left temporal parietal junction, posterior cingulate cortex bilaterally, and left medial orbitofrontal cortex. During the last time window (330–470 ms), however, the pattern of differences changed. Children expressed overall higher (*p*_adj_ = 0.004) PseudoZ activation compared with adults. These group differences were the most prominent in left parietal lobe and supplemental motor area, bilateral inferior temporal cortex, inferior frontal cortex, and medial orbitofrontal cortex.

### Whole-brain connectivity differences between children and adults

**Figure 6.**
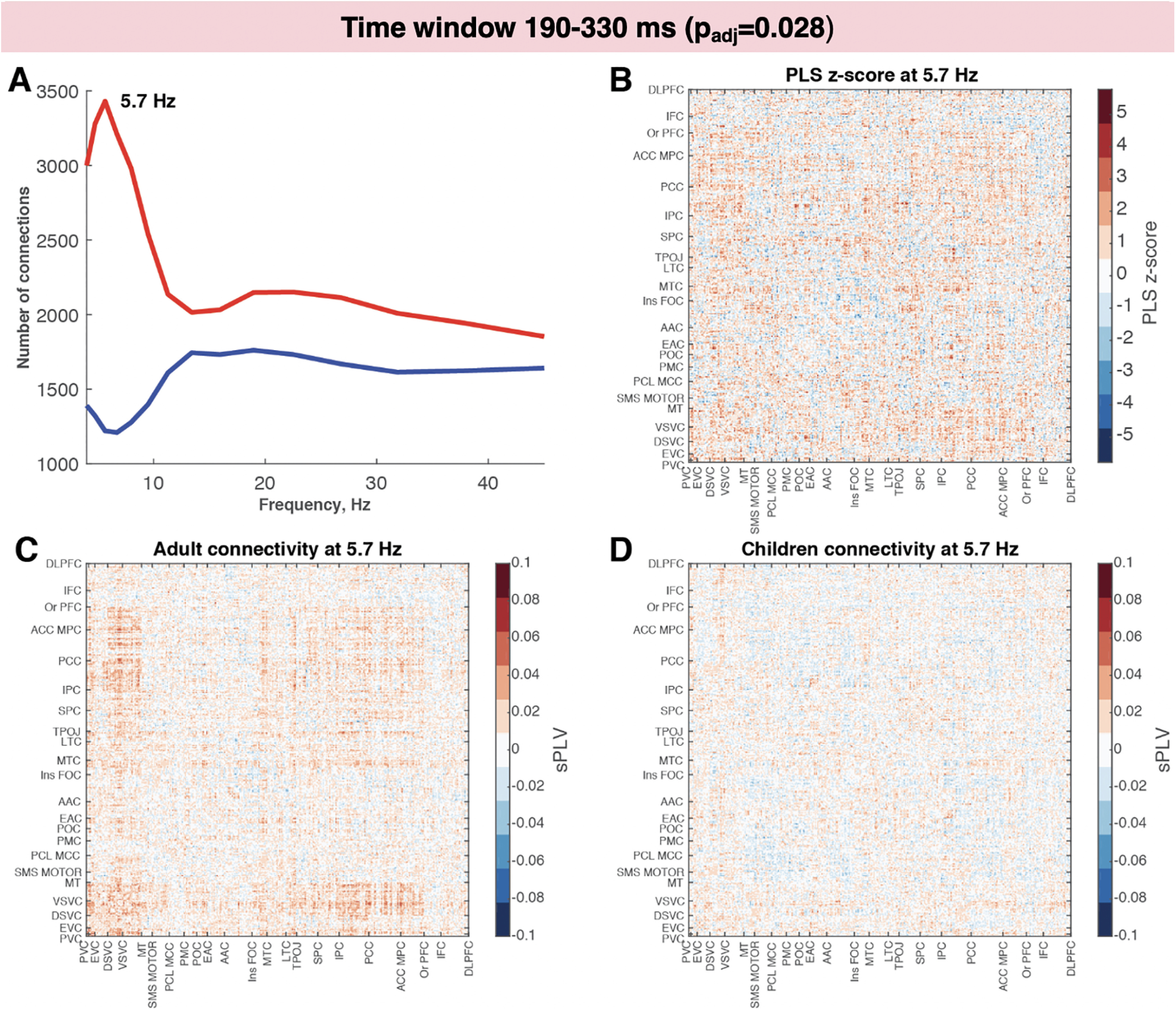
Increased intertrial synchrony in adults compared with children while viewing faces during the 190–330 ms time window. ***A***, Number of connections with PLS *z* score >2 (in red) or less than −2 (in blue) across frequency bins. ***B***, The *z* score distribution across connections for the frequency bin with the highest number of connections with *z* score above the selected threshold (5.7 Hz). ***C***, ***D***, Connectivity matrix for adults (***C***) and for children (***D***) in the same frequency bin.

To investigate interregional phase synchrony between cortical regions, we computed sPLVs for cortical ROIs defined in the MPA. Similar to the PseudoZ activation comparisons, we ran four PLS analyses (one for each time window). The results revealed significant differences between children and adults during two time windows: 120–190 ms (*p*_adj_ = 0.004; Fig. 5) and 190–330 ms (*p*_adj_ = 0.028; Fig. 6). For the first and the last time window, the sPLV was not different between children and adults (*p*_adj_ = 0.52 and *p*_adj_ = 0.8, respectively). To investigate which frequencies contributed the most to the group differences, we computed the number of connections with the lowest (< −2) and highest (>2) PLS *z* scores for each frequency bin (Figs. 5*A*, 6*A*). For both time windows, we observed the same pattern of increased connectivity in adults in lower-frequency bins (theta – lower alpha band) peaking at 6.7 Hz at 120–190 ms and 5.7 Hz at 190–330 ms. The spatial patterns were also similar between the two time windows. Higher-phase synchrony in adults was observed among visual areas including primary and early visual cortex, dorsal and ventral visual streams, and MT complex. These areas also exhibited increased connectivity with higher-order areas, including medial and lateral temporal cortices, TPOJ areas, parietal cortex, posterior and anterior cingulate cortices, and medial and orbital prefrontal cortices. The connectivity within these higher-order areas also was increased in adults compared with children especially during the second time window (120–190 ms).

### Control analyses

We conducted additional analyses using hand scenes (since this was the second largest category of movie scenes) to investigate whether differences in PseudoZ activation and phase synchronization were present only during face scenes. The motivation behind it was not to investigate hand–face contrast (the differences between face and hand conditions were extensively investigated in a previous study using a cohort of adult participants; Nunes et al., 2020), but to rule out the possibility of shared artifactual influences that would produce differences between children and adults. For example, if the differences in sPLV between children and adults were only because of the differential impact of volume conduction affecting sPLV estimation or the differences in PseudoZ activation were because of a lack of attention paid by children, such differences would not be condition specific (e.g., we would observe them during face conditions as well as hand conditions).

**Figure 7.**
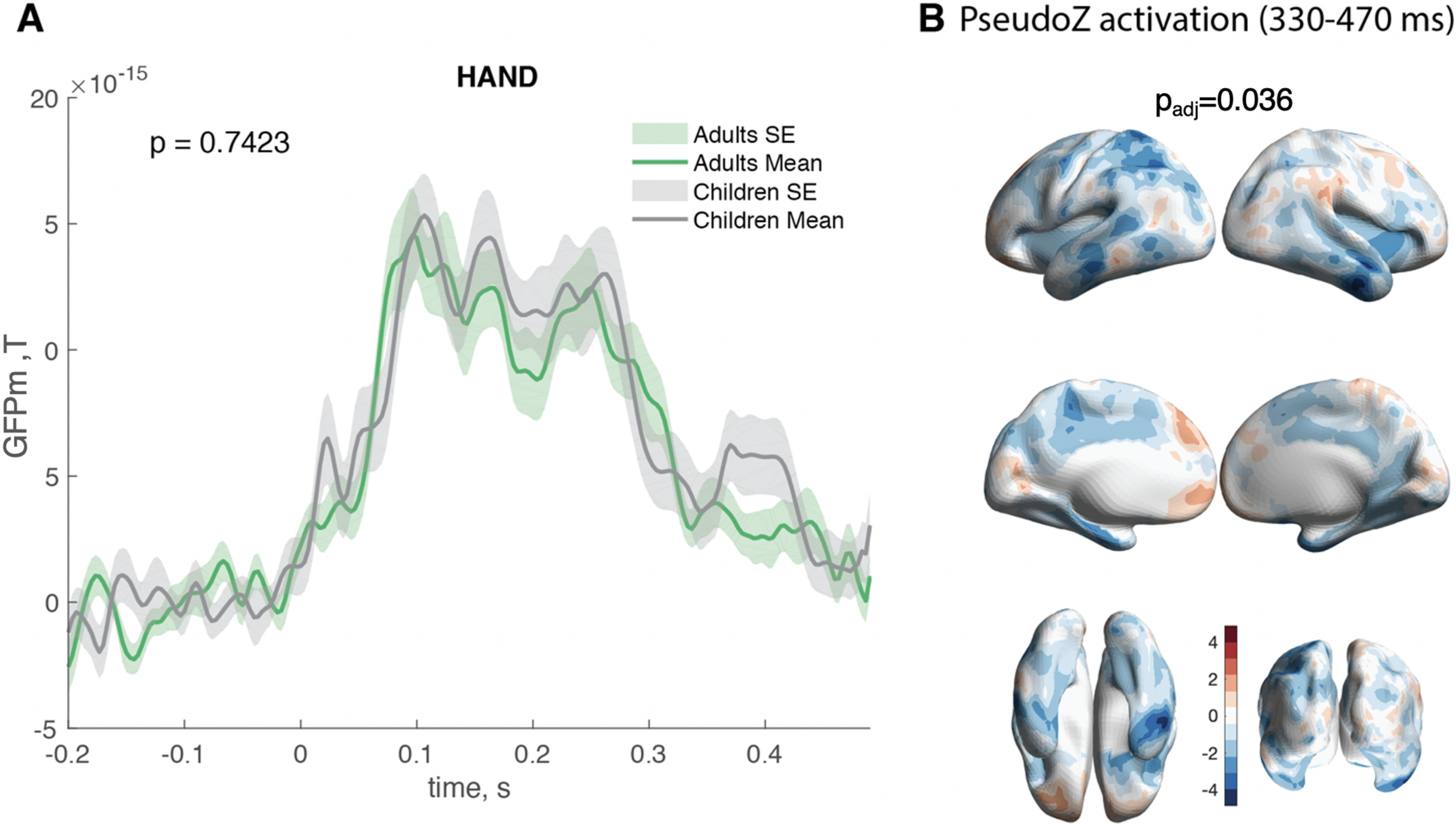
The control analysis of GFPm, PseudoZ activation, and interregional connectivity elicited by movie scenes with hands and hands manipulations between children and adults. ***A***, No significant differences in GFPm between children and adults (*p* = 0.74). GFPm in adults (in green) and children (in gray) averaged across all MEG channels and face epochs. The shadow represents the SE. ***B***, PLS *z* score distribution during the 330–470 ms time window reflecting significant group differences between children and adults during hand condition (*p*_adj_ = 0.036). Areas in red correspond to the positive *z* score, meaning higher activation in adults compared with children. Areas in blue correspond to negative *z* score and represent higher activation in children compared with adults.

Therefore, we repeated all steps of the analysis using epochs defined based on movie scenes displaying hands and hand manipulations in the foreground (58 in total). The comparison of GFPm was not significant between children and adults (*p* = 0.74; Fig. 7*A*). The comparison of PseudoZ activation, however, revealed significant differences between children and adults (*p*_adj_ = 0.036) for the last time window (330–470 ms; Fig. 6*B*), and the overall pattern of differences matched very closely the results revealed for the face condition in this time window (Fig. 3*C*). Therefore, we interpret differences for this time window for the main analysis with caution. The comparisons of PseudoZ activation during other time windows were not significant (50–120, 120–190, and 190–330 ms; *p*_adj_ < 0.4 for all). When we compared phase synchronization for hand epochs across the same ROIs, the connectivity during all time windows was not significantly different between children and adults (50–120, 120–190, 190–330, and 330–470 ms, *p*_adj_ < 0.75 for all).

## Discussion

In this study, we present novel findings on how face processing differs between children and adults in terms of whole-brain activation patterns and interregional phase locking elicited by faces during movie watching. Instead of a standard paradigm where simplistic face stimuli are presented repeatedly, our participants viewed a movie where scenes with faces in the foreground were used to define epochs of interest. Naturalistic stimuli allow to study face processing when other factors, common in day-to-day situations (e.g., natural speech, nonstatic facial expressions, and changing emotional status) are present. Because of potential interaction between primary face-processing areas and the rest of the cortex to process the complex stimuli, we conducted a large-scale whole-brain analysis of activation and connectivity rather than focusing on a subset of selected areas. Importantly, the employed source reconstruction technique was designed to highlight the activation was specific to scenes with faces and was not present during other scenes by using a noise covariance computed based on non-face scenes. Moreover, we investigated group differences using PLS analysis in the multivariate data-driven way with no statistical a priori assumption about a group contrast or data normality. Our results show that although children and adults exhibit similar activation patterns and interregional connectivity during early stages of face processing (50–120 ms), the later phases of face-elicited activation and synchronization are different. Adults expressed stronger activation and interregional connectivity compared with children. Spatially, the effects were observed in the core face-processing regions as well as higher-order areas involved in the extended face-processing network.

Although the sensor-based GFPm analysis across all MEG channels, detected significant differences between children and adults staring after 200 ms, the earliest significant difference in a spatially resolved analysis of face-related activation was observed between 120 and 190ms. While not significant in a univariate analysis of GFP across all sensors for this time-window, our PseudoZ results indicated that adults compared with children exhibited higher activation in occipital, inferior parietal, and middle and superior temporal cortices in the left hemisphere (Fig. 4*A*). The latency of this time window approximately corresponds to one of the most studied face-selective ERP components–the N170 that is believed to originate as a result the FG activation (for review, see Eimer, 2011). Note that this waveform is extensively studied by both EEG and MEG (referred to as M170) and has been shown to reflect similar cortical sources across both modalities (Deffke et al., 2007). Although typical right-hemisphere dominance of the FG is widely reported (Dehaene and Cohen, 2011; Li et al., 2013), cortical networks specializing in face processing are distributed across both hemispheres (Haxby and Gobbini, 2011). There is evidence that, in contrast to right FG, left hemispheric activation in FG is modulated by the contextual information surrounding face stimuli (Meng et al., 2012). Similarly, an ERP study that aimed to investigate the impact of the visual scene context on face processing found that a fearful context elicited higher amplitude of N170 in occipitotemporal sites of the left hemisphere (Righart and De Gelder, 2006). Given that our stimulus set consisted of scenes with faces during the movie presentation, these stimuli entailed a rich variation in face position, orientation, and the visual background surrounding faces and provided extra contextual information. Accordingly, it is likely that such left-hemispheric modulation might be less developed in children and reflected a lower posterior left-hemispheric activation in children compared with adults. Such an assumption, however, needs to be supported by future studies specifically designed to test this hypothesis.

The 190–330 ms time window after face onset revealed group differences in both PseudoZ activation and GFPm analysis. Adults had significantly higher GFPm of ∼250 ms, closely corresponding to the latency of the N250 (M250 in MEG) component. Interestingly, the N250 has been reported to be modulated by face repetition (that happens naturally during movie), reflecting the perceptual recognition of the individual stimuli (Schweinberger et al., 2002). A recent study demonstrated that the amplitude of the M250 was higher for repeated versus not-repeated faces in adults, but in 4- to 6-year-old children the amplitude was stable between the two conditions (He and Johnson, 2018). While such results are congruent with our finding of higher GFPm values at 230–270 ms in adults compared with children and might be linked to character recognition in the movie, future research will have to confirm face repetition as a main reason for such findings.

Group differences in PseudoZ activation included higher activation of the right pSTS, left temporal parietal junction, posterior cingulate cortex bilaterally, and left medial orbitofrontal cortex in adults compared with children. There is evidence that superior temporal gyrus is involved in the recognition of dynamic properties of facial expression (dorsal visual stream), while fusiform gyrus (ventral visual stream) is engaged in processing invariant facial aspects (Bernstein and Yovel, 2015). Activation in pSTS was shown to be modulated by facial expressions of emotion, speech-lip reading, and eye-gaze shifts (Schobert et al., 2018). Similarly, the comparison between static and dynamic facial expressions demonstrated enhanced activation of STS (Arsalidou et al., 2011). Behavioral research, however, indicates that while dynamic facial expressions reliably improve emotion recognition in adults, it does not have a similar effect on children between 4 and 10 years of age (Krumhuber et al., 2013; Widen and Russell, 2015). Speculatively, our findings of decreased activation of pSTS in children compared with adults could reflect the absence of dynamic face recognition that develops later on.

There is evidence that medial orbitofrontal cortex demonstrates robust face-selective activation (Troiani et al., 2016), which is modulated by emotional status, attractiveness, and social context of face stimuli (Pegors et al., 2015). Interestingly, PCC and TPOJ areas were shown to be activated when familiar faces were presented to the participants (Haxby and Gobbini, 2011). In addition, the PCC activation has been linked to empathy elicited by the viewing of emotional faces (Del Casale et al., 2017). Our results demonstrated that activation in these areas in children did not reach the adult level, which might explain previous behavioral findings of immaturity in these higher-level aspects of face processing (Decety, 2010; de Haan, 2011). Because of the limited number of scenes with faces, we were unable to further stratify face epoch based on their emotional content, but future studies should test this hypothesis by examining the emotional content of face stimuli in a movie.

Our findings also indicated that there were significant differences in cortical activation during the 330–450 ms time window. Children displayed a global pattern of higher widespread activation across the brain. Our control analysis (Fig. 7*B*), however, revealed that such overactivation was not specific to the face condition but was also present when the epochs were time locked to the appearance of hands on the screen. Thus, it might represent general overactivation elicited by the complex stimuli flow in children because of the fact that multiple neural mechanisms are still not fully matured and undergoing fine-tuning to increase specificity and efficiency via synaptic pruning (Benasich and Ribary, 2018).

Last, our results demonstrated that between 120 and 330 ms children and adults expressed differences in interregional connectivity. A clear pattern of higher connectivity in early visual cortex, dorsal and ventral visual streams, and MT-complex in slower frequencies (theta and lower alpha) was observed in adults and was not as evident in children. A recent MEG study reported organizational changes in effective connectivity structure among OFA, FG, and STS in the left and right hemispheres, and different modulation of connectivity strength elicited by face versus scrambled face stimuli in children and adults (He et al., 2015b). There are similar recent findings demonstrating that resting-state connectivity within the core face-processing network as well as task-based connectivity exhibit age-related increases (Kadosh et al., 2011; Haist et al., 2013; Song et al., 2015). The frequency specificity of our findings is also of interest. It has been shown that low-frequency oscillations in face-sensitive areas are modulated by motion and different face-related information such as emotional expression and identity (Popov et al., 2013; Furl et al., 2014; Güntekin and Başar, 2014). Theta synchronization has been shown to be enhanced by presentation of emotional faces (Knyazev et al., 2009). Another study demonstrated that low-frequency oscillations induced by dynamic faces encode identity, configural information, and motion of dynamic stimuli (Furl et al., 2017). Based on our findings of decreased theta–alpha connectivity in children compared with adults, we can hypothesize that such modulation by different aspects of facial-related information as well as motion is still undergoing developmental tuning in the age range of 10–12 years. Such an interpretation could be tested in the future by extending the age range to adolescence to track the connectivity change in this frequency range.

Our results of increased phase synchronization in adults also involved connections between visual areas and higher-order cortical regions including medial and lateral temporal cortex, TPOJ areas, parietal cortex, posterior and anterior cingulate cortices, and medial and orbital prefrontal cortices. The connectivity involving these higher-order areas was also increased in adults compared with children. Previously, two fMRI studies used connectivity analysis to extend their regions of interest beyond the core face network. However, their results were somewhat contradictory (but not mutually exclusive), with one study reporting higher connectivity in adults compared with children (Joseph et al., 2012), whereas another reported a negative correlation between age and connectivity between the core and extended network (Wang et al., 2018). Speculatively, given the nature of our stimuli, such an interaction is needed to support the integration of incoming information about face emotion, identity, and context within the movie. This suggestion is based on multiple lines of evidence indicating the importance of the extended face-processing network in addition to core face-selective regions in higher-level aspects of face processing. Those include conscious representations of emotional states, behavior regulation in social situations, reading intentions, and predicting future actions (de Haan, 2011). Such functions are supported by coordinated activity among anterior temporal cortex, TPJ areas, precuneus, inferior frontal gyrus, medial prefrontal and orbitofrontal cortices, and anterior cingulate cortex (Haxby et al., 2000; Gobbini and Haxby, 2007). Efficient communication among these regions is needed for the extraction and processing of plentiful information secluded in naturalistic faces.

Our study is not without limitations. Although movie viewing is widely considered to better mimic real-life stimuli compared with standard paradigms, it still differs from the real-life experience in multiple respects that could affect the perception and brain processing, and this should be taken into account. Since our aim was to investigate face processing using a novel complex paradigm, we used an exploratory rather than a hypothesis-driven approach. While being advantageous for generating new data-driven results that can be used for setting specific hypotheses, our results should be confirmed by future studies. Since no standard face stimuli paradigm data were available for our cohort, we were unable to conduct a direct comparison of developmental differences based on the movie and standard face stimuli. Therefore, our attempt to contextualize our findings with respect to already existing reports should be interpreted with caution. Last, the limited sample size means that further replication of these results is warranted, and that other potential developmental effects associated with face processing may not have been detectable with this sample size and should be probed by future studies with larger cohorts.

Overall, our findings provide important new insights on how face processing differs between children and adults within the dynamic naturalistic context of a movie setting. It supports and extends the existing evidence of profound maturational changes in both a core face-processing network and an extended system of higher-order cortical areas. Notably, we used a naturalistic paradigm to investigate face processing in a time-locked manner that allowed us to preserve the ecological validity of the stimuli while still focusing on face-related neural processes. Our results further support the possibility of exploiting naturalistic stimuli to investigate specific questions regarding sensory processing as an alternative to standard paradigms.

## Synthesis

Reviewing Editor: Christoph Michel, Universite de Geneve Decisions are customarily a result of the Reviewing Editor and the peer reviewers coming together and discussing their recommendations until a consensus is reached. When revisions are invited, a fact-based synthesis statement explaining their decision and outlining what is needed to prepare a revision will be listed below. The following reviewer(s) agreed to reveal their identity: Nicholas Furl.

Despite the editor’s request, the authors declined to add new analyses.

Three new analysis were suggested by the editor based on the reviewers comments:

1. A comparison of face vs. hand scenes to claim for face-specificity of the identified network.

2. A direct comparison of standardized static face pictures with faces in movie scenes to support the claim that differences between children and adults are similar to what has previously been described for standardized face pictures.

3. A statistical comparison between left and right hemisphere to support the claim of stronger activation in the right hemisphere.

In the rebuttal letter the authors gave the following arguments why these additional analyses were not performed.

1. Face-specificity was assured by the fact that the PseudoZ calculation of the source reconstruction includes different non-face scenes as contrast to compute the noise-covariance.

2. No standard face picture paradigm data was available. They explicitly mention this in the limitation section

3. The claim of hemispheric asymmetry was removed.

The Answers to point 1 indeed clarifies the face-specificity claim. We recommend that the fact that the baseline of the source reconstruction includes hands and other non-face content is verbalised more prominently in the paper. It should not be buried in one line in the Methods because it’s a key fact needed for interpretation of the source reconstruction results. We ask the authors to lay this point out more clearly at least in the Abstract and Discussion.

Concerning point 2, we understand that conducting a new experiment would have required substantial work. However, in our synthesis statement we suggested to perform a traditional evoked univariate analyses of the standard components like M100 and M170 and qualitatively compare these results with those of previous literature on static images instead of only performing the PLS analysis. This would address the question “Do naturalistic movie scenes reproduce the face selectivity on the M170 commonly seen for static images?”. We think such an analysis is feasible and would strengthen the claim that the observed differences between children and adults are similar to what has been described for standardized pictures.

Concerning point 3, the elimination of claims on lateralization is acceptable.

One additional request concerns clarifications with respect to the PLS analysis. The sentence: “There is no need to specify a predefined group contrast when using the mean-centered PLS as it generates a data-driven group contrast fully based on present data.” is not clear. The PLS user decides what the groups are to be contrasted by how they construct the matrix going to the SVD. So in what sense is the contrast not pre-specified? Plus, it is not clear what “it generates a data-driven group contrast” means. Last: why is NOT prespecifying a contrast an advantage? If the authors have a hypothesis about differing age groups than an analysis that allows one to specify a contrast of age groups is what is advantageous. The rest of the paper would be perfectly interpretable without this confusing description.

## References

[B1] Arsalidou M, Morris D, Taylor MJ (2011) Converging evidence for the advantage of dynamic facial expressions. Brain Topogr 24:149–163. 10.1007/s10548-011-0171-4 21350872

[B2] Benasich A, Ribary U (2018) Emergent brain dynamics: prebirth to adolescence. Cambridge, MA: MIT.

[B3] Bernstein M, Yovel G (2015) Two neural pathways of face processing: a critical evaluation of current models. Neurosci Biobehav Rev 55:536–546. 10.1016/j.neubiorev.2015.06.010 26067903

[B4] de Haan M (2011) The neurodevelopment of face perception. In: The Oxford handbook of face perception (Calder A, Rhodes G, Johnson M, Haxby J, eds), pp 934–961. Oxford, UK: Oxford UP.

[B5] Decety J (2010) The neurodevelopment of empathy in humans. Dev Neurosci 32:257–267. 10.1159/000317771 20805682 PMC3021497

[B6] Deffke I, Sander T, Heidenreich J, Sommer W, Curio G, Trahms L, Lueschow A (2007) MEG/EEG sources of the 170-ms response to faces are co-localized in the fusiform gyrus. Neuroimage 35:1495–1501. 10.1016/j.neuroimage.2007.01.034 17363282

[B7] Dehaene S, Cohen L (2011) The unique role of the visual word form area in reading. Trends Cogn Sci 15:254–262. 10.1016/j.tics.2011.04.003 21592844

[B8] Del Casale A, Kotzalidis GD, Rapinesi C, Janiri D, Aragona M, Puzella A, Spinazzola E, Maggiora M, Giuseppin G, Tamorri SM, Vento AE, Ferracuti S, Sani G, Pompili M, Girardi P (2017) Neural functional correlates of empathic face processing. Neurosci Lett 655:68–75. 10.1016/j.neulet.2017.06.058 28673832

[B9] Eimer M (2011) The face-sensitive N170 component of event-related brain potential. In: The Oxford handbook of face perception (Calder AJ, Rhodes G, Johnson MH, Haxby JV, eds), pp 329–345. Oxford, UK: Oxford UP.

[B10] Fischl B, Sereno MI, Dale AM (1999) Cortical surface-based analysis: II. Inflation, flattening, and a surface-based coordinate system. Neuroimage 9:195–207. 10.1006/nimg.1998.0396 9931269

[B11] Furl N, Coppola R, Averbeck BB, Weinberger DR (2014) Cross-frequency power coupling between hierarchically organized face-selective areas. Cereb Cortex 24:2409–2420. 10.1093/cercor/bht097 23588186 PMC4128705

[B12] Furl N, Lohse M, Pizzorni-Ferrarese F (2017) Low-frequency oscillations employ a general coding of the spatio-temporal similarity of dynamic faces. Neuroimage 157:486–499. 10.1016/j.neuroimage.2017.06.023 28619657 PMC6390175

[B13] Glasser MF, Coalson TS, Robinson EC, Hacker CD, Harwell J, Yacoub E, Ugurbil K, Andersson J, Beckmann CF, Jenkinson M, Smith SM, Van Essen DC (2016) A multi-modal parcellation of human cerebral cortex. Nature 536:171–178. 10.1038/nature18933 27437579 PMC4990127

[B14] Gobbini MI, Haxby JV (2007) Neural systems for recognition of familiar faces. Neuropsychologia 45:32–41. 10.1016/j.neuropsychologia.2006.04.015 16797608

[B15] Greene DJ, Koller JM, Hampton JM, Wesevich V, Van AN, Nguyen AL, Hoyt CR, McIntyre L, Earl EA, Klein RL, Shimony JS, Petersen SE, Schlaggar BL, Fair DA, Dosenbach NUF (2018) Behavioral interventions for reducing head motion during MRI scans in children. Neuroimage 171:234–245. 10.1016/j.neuroimage.2018.01.023 29337280 PMC5857466

[B16] Güntekin B, Başar E (2014) A review of brain oscillations in perception of faces and emotional pictures. Neuropsychologia 58:33–51. 10.1016/j.neuropsychologia.2014.03.014 24709570

[B17] Haist F, Adamo M, Han Wazny J, Lee K, Stiles J (2013) The functional architecture for face-processing expertise: FMRI evidence of the developmental trajectory of the core and the extended face systems. Neuropsychologia 51:2893–2908. 10.1016/j.neuropsychologia.2013.08.005 23948645 PMC3825803

[B18] Haxby JV, Gobbini IM (2011) Distributed neural systems for face perception. In: The Oxford handbook of face perception (Calder A, Rhodes G, Johnson M, Haxby J, eds), pp 93–110. Oxford, UK: Oxford UP.

[B19] Haxby JV, Hoffman EA, Gobbini MI (2000) The distributed human neural system for face perception. Trends Cogn Sci 4:223–233. 10.1016/s1364-6613(00)01482-0 10827445

[B20] He W, Brock J, Johnson BW (2015a) Face processing in the brains of pre-school aged children measured with MEG. Neuroimage 106:317–327. 10.1016/j.neuroimage.2014.11.029 25463467

[B21] He W, Garrido MI, Sowman PF, Brock J, Johnson BW (2015b) Development of effective connectivity in the core network for face perception. Hum Brain Mapp 36:2161–2173. 10.1002/hbm.22762 25704356 PMC6869188

[B22] He W, Johnson BW (2018) Development of face recognition: dynamic causal modelling of MEG data. Dev Cogn Neurosci 30:13–22. 10.1016/j.dcn.2017.11.010 29197727 PMC6969123

[B23] Joseph JE, Gathers AD, Bhatt RS (2011) Progressive and regressive developmental changes in neural substrates for face processing: testing specific predictions of the Interactive Specialization account. Dev Sci 14:227–241. 10.1111/j.1467-7687.2010.00963.x 21399706 PMC3050484

[B24] Joseph JE, Swearingen JE, Clark JD, Benca CE, Collins HR, Corbly CR, Gathers AD, Bhatt RS (2012) The changing landscape of functional brain networks for face processing in typical development. Neuroimage 63:1223–1236. 10.1016/j.neuroimage.2012.08.021 22906788 PMC3637657

[B25] Joseph JE, Vanderweyen D, Swearingen J, Vaughan BK, Novo D, Zhu X, Gebregziabher M, Bonilha L, Bhatt R, Naselaris T, Dean B (2019) Tracking the development of functional connectomes for face processing. Brain Connect 9:231–239. 10.1089/brain.2018.0607 30489152 PMC6444905

[B26] Kadosh KC, Kadosh RC, Dick F, Johnson MH (2011) Developmental changes in effective connectivity in the emerging core face network. Cereb Cortex 21:1389–1394. 10.1093/cercor/bhq215 21045001 PMC3094719

[B27] Kanwisher N, Yovel G (2006) The fusiform face area: a cortical region specialized for the perception of faces. Philos Trans R Soc Lond B Biol Sci 361:2109–2128. 10.1098/rstb.2006.1934 17118927 PMC1857737

[B28] Knyazev GG, Slobodskoj-Plusnin JY, Bocharov AV (2009) Event-related delta and theta synchronization during explicit and implicit emotion processing. Neuroscience 164:1588–1600. 10.1016/j.neuroscience.2009.09.057 19796666

[B29] Kozhemiako N, Nunes A, Vakorin VA, Chau CMY, Moiseev A, Ribary U, Grunau RE, Doesburg SM (2019) Atypical resting state neuromagnetic connectivity and spectral power in very preterm children. J Child Psychol Psychiatr 60:975–987. 10.1111/jcpp.13026 30805942

[B30] Kozhemiako N, Nunes AS, Vakorin VA, Chau CMY, Moiseev A, Ribary U, Grunau RE, Doesburg SM (2020) Sex differences in brain connectivity and male vulnerability in very preterm children. Hum Brain Mapp 41:388–400. 10.1002/hbm.24809 31587465 PMC7267928

[B31] Krishnan A, Williams LJ, McIntosh AR, Abdi H (2011) Partial least squares (PLS) methods for neuroimaging: a tutorial and review. Neuroimage 56:455–475. 10.1016/j.neuroimage.2010.07.034 20656037

[B32] Krumhuber EG, Kappas A, Manstead ASR (2013) Effects of dynamic aspects of facial expressions: a review. Emot Rev 5:41–46. 10.1177/1754073912451349

[B33] Li S, Lee K, Zhao J, Yang Z, He S, Weng X (2013) Neural competition as a developmental process: early hemispheric specialization for word processing delays specialization for face processing. Neuropsychologia 51:950–959. 10.1016/j.neuropsychologia.2013.02.006 23462239 PMC3756286

[B34] Marzi T, Viggiano MP (2007) Interplay between familiarity and orientation in face processing: an ERP study. Int J Psychophysiol 65:182–192. 10.1016/j.ijpsycho.2007.04.003 17512996

[B35] McIntosh AR, Lobaugh NJ (2004) Partial least squares analysis of neuroimaging data: applications and advances. Neuroimage 23:S250–S263. 10.1016/j.neuroimage.2004.07.020 15501095

[B36] Meng M, Cherian T, Singal G, Sinha P (2012) Lateralization of face processing in the human brain. Proc Biol Sci 279:2052–2061. 10.1098/rspb.2011.1784 22217726 PMC3311882

[B37] Nunes AS, Kozhemiako N, Moiseev A, Seymour RA, Cheung TPL, Ribary U, Doesburg SM (2020) Neuromagnetic activation and oscillatory dynamics of stimulus-locked processing during naturalistic viewing. Neuroimage 216:116414. 10.1016/j.neuroimage.2019.116414 31794854

[B38] Oostenveld R, Fries P, Maris E, Schoffelen J-M (2011) FieldTrip: open source software for advanced analysis of MEG, EEG, and invasive electrophysiological data. Comput Intell Neurosci 2011:156869. 10.1155/2011/156869 21253357 PMC3021840

[B39] Pegors TK, Kable JW, Chatterjee A, Epstein RA (2015) Common and unique representations in pFC for face and place attractiveness. J Cogn Neurosci 27:959–973. 10.1162/jocn_a_00777 25539044 PMC4681394

[B40] Popov T, Miller GA, Rockstroh B, Weisz N (2013) Modulation of α power and functional connectivity during facial affect recognition. J Neurosci 33:6018–6026. 10.1523/JNEUROSCI.2763-12.2013 23554483 PMC6618940

[B41] Puce A, McNeely ME, Berrebi ME, Thompson JC, Hardee J, Brefczynski-Lewis J (2013) Multiple faces elicit augmented neural activity. Front Hum Neurosci 7:282. 10.3389/fnhum.2013.00282 23785327 PMC3682123

[B42] Righart R, De Gelder B (2006) Context influences early perceptual analysis of faces: an electrophysiological study. Cereb Cortex 16:1249–1257. 10.1093/cercor/bhj066 16306325

[B43] Sasson NJ (2006) The development of face processing in autism. J Autism Dev Disord 36:381–394. 10.1007/s10803-006-0076-3 16572261

[B44] Schobert AK, Corradi-Dell’Acqua C, Frühholz S, van der Zwaag W, Vuilleumier P (2018) Functional organization of face processing in the human superior temporal sulcus: a 7T high-resolution fMRI study. Soc Cogn Affect Neurosci 13:102–113. 10.1093/scan/nsx119 29140527 PMC5793830

[B45] Schweinberger SR, Pickering EC, Jentzsch I, Burton AM, Kaufmann JM (2002) Event-related brain potential evidence for a response of inferior temporal cortex to familiar face repetitions. Brain Res Cogn Brain Res 14:398–409. 10.1016/s0926-6410(02)00142-8 12421663

[B46] Song Y, Zhu Q, Li J, Wang X, Liu J (2015) Typical and atypical development of functional connectivity in the face network. J Neurosci 35:14624–14635. 10.1523/JNEUROSCI.0969-15.2015 26511251 PMC6605463

[B47] Taylor MJ, Batty M, Itier RJ (2004) The faces of development: a review of early face processing over childhood. J Cogn Neurosci 16:1426–1442. 10.1162/0898929042304732 15509388

[B48] Troiani V, Dougherty CC, Michael AM, Olson IR (2016) Characterization of face-selective patches in orbitofrontal cortex. Front Hum Neurosci 10:279. 10.3389/fnhum.2016.00279 27378880 PMC4906043

[B49] Vanderwal T, Kelly C, Eilbott J, Mayes LC, Castellanos FX (2015) Inscapes: a movie paradigm to improve compliance in functional magnetic resonance imaging. Neuroimage 122:222–232. 10.1016/j.neuroimage.2015.07.069 26241683 PMC4618190

[B50] Wang X, Zhu Q, Song Y, Liu J (2018) Developmental reorganization of the core and extended face networks revealed by global functional connectivity. Cereb Cortex 28:3521–3530. 10.1093/cercor/bhx217 28968833

[B51] Widen SC, Russell JA (2015) Do dynamic facial expressions convey emotions to children better than do static ones? J Cogn Dev 16:802–811. 10.1080/15248372.2014.916295

[B52] Wieser MJ, Brosch T (2012) Faces in context: a review and systematization of contextual influences on affective face processing. Front Psychol 3:471. 10.3389/fpsyg.2012.00471 23130011 PMC3487423

[B53] Young AW, Burton AM (2018) Are we face experts? Trends Cogn Sci 22:100–110. 10.1016/j.tics.2017.11.007 29254899

